# Haptenization as the missing link between vasculitis and myeloperoxidase

**DOI:** 10.1172/JCI191587

**Published:** 2025-04-15

**Authors:** Laura Santambrogio

**Affiliations:** Weill Cornell Medicine, New York, New York, USA.

## Abstract

A wide variety of medications can induce adverse immune events and autoimmune responses such as vasculitis. Mechanistically, small molecule drugs known as haptens bind and modify endogenous proteins, triggering such immune reactions. In this issue of the *JCI*, Xi and colleagues investigated the immunological mechanism of autoimmune vasculitis associated with hydralazine. Notably, hydralazine-based haptenization modified myeloperoxidase (MPO), inducing the enzyme conformational change. The hydralazine-modified MPO induced IgM antibody specific for the modified enzyme, followed by immune complex precipitation, tissue deposition, and complement activation. These findings provide a mechanism by which hydralazine induces a type III hypersensitivity reaction associated with mild to severe vasculitis. The study serves as an example for understanding haptenation and may inform the development of diagnostics for determining susceptibility to drug-induced allergic or autoimmune responses.

## Direct and indirect haptenization

Drug-induced immune-related adverse events are a raising cause of immune reactions triggered by a wide variety of medications, including antibiotics, cardiovascular medications such as statins and calcium antagonists, cancer medications such as aromatase inhibitors and immune checkpoint inhibitors, and anticonvulsant drugs, among many others. Autoimmune diseases that have been linked to drug-induced immune autoreactivity include: lupus erythematosus, rheumatoid arthritis, polymyositis, dermatomyositis, myasthenia gravis, pemphigus, membranous glomerulonephritis, autoimmune hepatitis, thyroiditis, and vasculitis.

Mechanistically, most of these immune reaction–inducing drugs function as haptens; small molecules (under 1,000 daltons) that can only elicit an immune response when bound to proteins by directly modifying their structure. The concept of hapten was developed almost 100 years ago by Karl Landsteiner (1), and, over the years, many examples of small molecules functioning as haptens have been described (2, 3).

At the chemical level, a prehapten molecule needs to be chemically activated to acquire sensitizing potential. The specific chemistry for prehapten activation involves direct oxidation by metal ions or free radicals, photoactivation by UV light, or enzymatic activation by a variety of enzymes, including the cytochrome P450 system, peroxidases, or oxygenases, to name few (4). Once activated, the prehaptens achieve an electrophilic state, defined as an atom or a molecule deficient in electrons, conducive for haptenization. Such molecules are positively charged and interact with an electron donor or nucleophile to achieve chemical stability through covalent binding (4). A second possibility for haptenization occurs as the proteins themselves achieve an electrophilic state through reactive species present in conditions of oxidative and metabolic stress (5–7). Once reactive, the protein can be directly haptenized.

In either case scenario, the direct or indirect haptenization will modify the protein primary and sometimes secondary, tertiary, and quaternary structures. More importantly, the newly generated hapten-modified protein will potentially lose its immunological self-privilege status and possibly elicit an autoreactive immune response (5–7).

## Hydralazine as a prehapten in autoimmune vasculitis

In this issue of the *JCI*, Xi et al. (8) report on the mechanism of a long-known autoimmune vasculitis associated with hydralazine exposure (9). Hydralazine is a commonly used vasodilator administrated orally to treat hypertension and intravenously to rapidly reduce blood pressure in medical emergencies such as eclampsia. Hydralazine has long been known to induce an autoimmune vasculitis in 5% to 10% of patients, depending on the dose and length of treatment (10), but the immunological mechanism was not yet fully elucidated.

Herein, the authors report that hydralazine-modified myeloperoxidase (MPO) constituted an autoimmune target to circulating IgM in a cohort of patients (8). The IgM bind with high affinity to hydralazine-MPO and induced a type-III hypersensitivity reaction, resulting in acute vasculitis and tissue damage (8).

**Figure 1:**
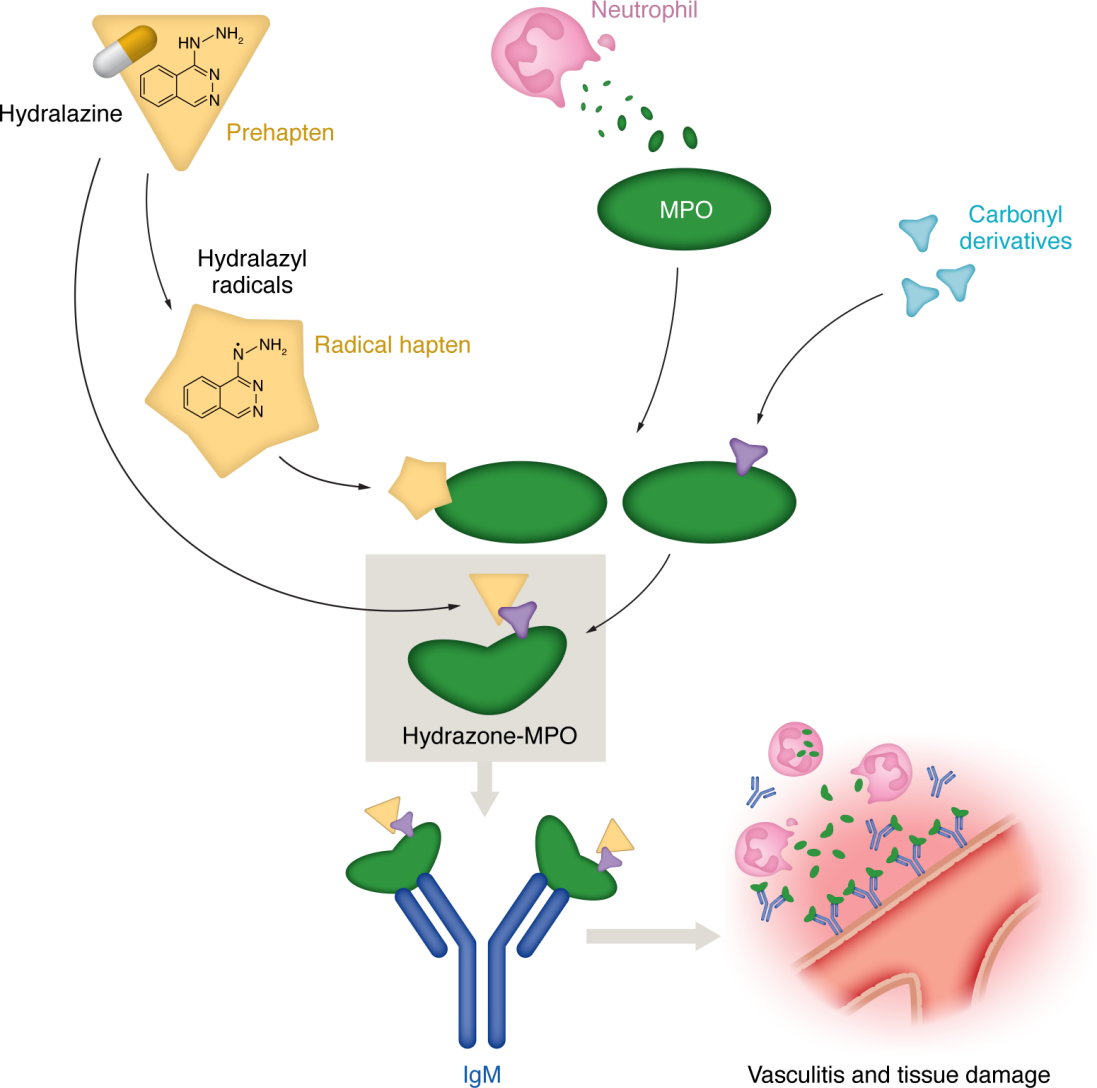
The mechanism of Hydralazine-induced vasculitis involves IgM reactivity to hydralazine-modified MPO. Hydralazine may function as a prehapten, from which enzymatic reaction or direct oxidation generates radical haptens, for example, hyralazyl radicals, that readily bind MPO. Alternatively, hydralazine can bind to MPO by linking through carbonyl derivatives already found on the protein itself. One of the molecules generated through the carbonyl-modified haptenization process is hydrazone-MPO, which induces anti-IgM specific for the modified, but not native, MPO. The IgM generates an antineutrophil cytoplasmic antibody (ANCA) response and neutrophil degranulation. IgM-bound hydrazone-MPO immune complexes induce a type III hypersensitivity reaction and activate the classical complement pathway, generating vasculitis and tissue damage.

Hydralazine may function as a prehapten, and several hydralazine radicals are known to be generated through metal, oxygen, or hypochlorous acid–mediated oxidation. Alternatively, hydralazine radicals are generated by enzymatic activation, such as with acetylases (i.e. acetylhydrazinophthalazinone), CYP450, and myeloperoxidase (phthalazinone), and others (11). These hydralazine radicals will then form a covalent bond with proteins generating hydralazine-radical–modified molecules. At the same time, there is a second possibility in which proteins exposed to oxidative and metabolic stress are modified by highly reactive carbonyl groups that promote a direct binding to hydralazine. In either case the final outcome is the generation of proteins haptenized by hydralazine-derived adducts (Figure 1). In Xi et al. (8), the authors used mass spectrometry to identify hydrazone adducts on both histones and MPO in individuals diagnosed with autoimmune vasculitis (Figure 1).

When proteins are haptenized, two major outcomes may occur: (a) the hapten groups induce an irreversible protein unfolding, aggregation, and loss of function, thus targeting the protein for degradation, or (b) the protein partially or wholly maintains its functionality, depending on where the hapten groups are located, but changes its primary structure and overall conformation . The latter is the outcome observed in Xi et al. (8) Hydralazine radical–modified MPO underwent a conformational change, as probed by a series of anti-MPO antibodies, which could only recognize the native MPO conformation but not the one modified by hydrazone adducts. Likely, the hydrazone groups either generated a steric hindrance to the antibody binding site, or they altogether induced a different MPO conformation, which masked the antibody-binding epitope. To further this point, Xi and authors showed that anti-hydralazine–MPO antibody could only immunoprecipitate MPO in patients with hydralazine-associated vasculitis (8).

The authors went on to characterize the MPO-hydrazone modifications as constituting an autoimmune target of circulating IgM. Albeit anti-IgG and -IgM antibodies to MPO were present in all individuals with antineutrophil cytoplasmic antibody–associated (ANCA-associated) vasculitis, the increase in anti-MPO IgM in this patient cohort was higher compared with patients with nonhydralazine-mediated vasculitis. More importantly, IgM — affinity purified from these participants — could react with hydralazine-modified MPO and induce neutrophil degranulation. Furthermore, the authors determined that glomerulonephritis could be induced by injecting an antibody targeting hydralazine-modified MPO (8).

In the early 1960, Philip Gell and Robert Coombs described four possible outcomes associated with hypersensitivity reactions (12, 13). IgE atopic reactions with mast cells and basophils degranulation (type I); antibody-mediated cellular cytotoxicity (IgM or IgG) (type II); immune complex precipitation, with tissue deposition and complement activation (type III); and T cell hypersensitivity responses in which hapten-modified self peptides are presented by major histocompatibility complex molecules (type IV) (14).

The hydralazine-mediated hypersensitivity reported in Xi et al. (8) was a classical type III hypersensitivity reaction, generated by an increased formation of IgM antibody specific to the hydrazone-MPO. Once generated, the IgM-hydrazone MPO circulating immunocomplexes precipitate and deposit in different tissues such as skin, joints, vessels, or glomeruli and trigger activation of the classical complement pathway (Figure 1). Complement activation results in inflammatory cell recruitment accompanied by the release of lysosomal enzymes and free radicals at areas of immune complex deposit, causing further tissue damage. Indeed, symptoms of hydralazine-induced vasculitis comprise a wide variety of clinical signs depending on where the IgM hydrazone-MPO complexes are deposited, including skin rashes with hives, bruising, and the presence of edema, joint inflammation, numbness or tingling due to nerve damage, fever, and, in severe cases, the vasculitis can lead to severe organ damage in the kidneys, heart, and lungs. As in all autoimmune responses, genetic predispositions and individual variations in immune responses play a role in determining why only a small percentage of individuals exposed to hydralazine will develop immune complex–associated vasculitis (15, 16).

The vasculitis described in Xi et al. (8) is part of a larger category of ANCA-associated vasculitis, which comprise Wegener’s granulomatosis, Churg-Strauss syndrome, microscopic polyangiitis, and renal limited vasculitis, all of which are idiopathic autoimmune diseases. On the other hand, it has recently been recognized that drugs from almost every pharmacological class can be implicated in causing vasculitis and a raising subcategory are associated with ANCA. Indeed, up to 20% of vasculitis cases have been associated with drug intake, with people over 50 years old being more susceptible (17).

## Conclusions and clinical implications

Altogether, Xi et al. (8) sheds light on the mechanism by which hydralazine, a widely prescribed medication, can induce a type III hypersensitivity reaction associated with mild to severe vasculitis.

The increasing number of cases in which chemicals or drugs behave as haptens in inducing allergic and autoimmune responses highlights the critical necessity of considering medication-induced complications in the clinic every time hypersensitivity reactions are observed. At the same time, in the laboratory, the development of additional testing for evaluating, in advance, whether a drug could cause allergic and autoimmune responses is warranted.

## Version 1 04/15/2025

Electronic publication
